# Asymmetric Osteotomies in Structural Rhinoplasty: A Pisa Tower-Inspired Approach for Crooked Noses

**DOI:** 10.1093/asjof/ojag064

**Published:** 2026-04-11

**Authors:** Nicola Bianco, Antonio Varricchio, Davide De Cicco, Annachiara Cavaliere, Daniele Lizambri, Santolo D’Antonio

## Abstract

**Background:**

The Pisa Tower Concept, originally described by Finocchi et al, corrects bony vault deviation through asymmetric osteotomies without dorsal reduction. This study evaluates a structural adaptation in which asymmetric osteotomies are integrated into a structural workflow including dorsal hump management and septal reconstruction.

**Objectives:**

The objective of this study was to assess the effectiveness, safety, and satisfaction of Pisa Tower–style osteotomies as an adjunct to structural rhinoplasty for crooked nose.

**Methods:**

A retrospective analysis was conducted on 37 patients undergoing structural rhinoplasty for crooked nose deformity. Osteotomies were adapted from the Pisa Tower model: low-to-low or high-to-low lateral osteotomy on the concave side, wedge resection on the convex side, and transverse or oblique osteotomy depending on deviation origin. All patients underwent endoscopic L-strut septoplasty, dorsal hump removal, and tip support with a septal extension graft. Outcomes included pre- and postoperative CT-based R–Webster angles, nasal axis deviation, a 5-point Likert satisfaction scale, and complications, after a mean follow-up of 18 months (range 12-28).

**Results:**

Mean nasal axis deviation improved from 7.9° ± 1.9° to 1.2° ± 0.6° (*P* = .0001). R–Webster distance on the convex side decreased from 30.4 ± 4.8 to 23.1 ± 3.9 mm (*P* = .0003); concave side, from 23.7 ± 3.5 to 22.8 ± 3.3 mm (*P* = .18). Overall, 89.2% of patients rated satisfaction as 4 or 5. Minor complications occurred in 13.5%; none were major.

**Conclusions:**

In this preliminary report, Pisa Tower–style asymmetric osteotomies may represent a promising adjunct to structural rhinoplasty for the correction of bony nasal deviation, extending Finocchi's concept to a structural framework.

**Level of Evidence: 4 (Therapeutic):**

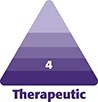

Correction of the crooked nose remains one of the most technically demanding aspects of rhinoplasty due to complex 3-dimensional deformities involving both the bony and cartilaginous framework, often associated with functional compromise.^1^ Achieving symmetry and stability requires precise mobilization of the bony and cartilaginous framework.^2^ Crooked nose deformities can be classified based on the axis of deviation, such as I-shaped (linear deviation), C-shaped (concave–convex asymmetry), and S-shaped (complex multilevel deviation), each requiring tailored surgical correction strategies.^1^

The Pisa Tower Concept, introduced by Finocchi et al, utilizes asymmetric osteotomies—with a “push-down” of the concave side and wedge resection of the convex side—to correct nasal deviation through en bloc mobilization of the bony vault.^3^ This technique was conceived within the framework of preservation rhinoplasty, where the dorsal lines remain intact and dorsal hump reduction is avoided.

In contrast, the present study explores a *structural adaptation* of that geometric rationale. The dorsal hump is reduced, the septum reconstructed under endoscopic vision, and tip support restored with a septal extension graft. To clearly differentiate it from Finocchi's original preservation-based approach, we refer to this modification as the Pisa Tower–style structural rhinoplasty.^3-5^ This study aimed to assess the feasibility and outcomes of applying Pisa Tower–style asymmetric osteotomies in structural rhinoplasty for crooked noses.

## METHODS

### Study Design and Population

A retrospective single-surgeon study was conducted on patients operated between January 2014 and December 2022 for primary rhinoplasty to correct crooked nose deformities, using a standardized operative approach based on the Structural Pisa Tower technique.

Patients were deemed eligible for analysis according to the following inclusion criteria: (1) diagnosis of a crooked nose deformity, (2) surgical correction performed using the Structural Pisa Tower technique, (3) procedure executed personally by the senior author (N.B.), and (4) operation performed within the specified time frame.

An initial screening of the database was conducted by 1 of the authors (S.D.A.), who identified all potentially eligible cases in accordance with the inclusion criteria. A subsequent eligibility review was jointly performed by 2 authors (S.D.A. and N.B.), according to the following exclusion criteria: (1) incomplete clinical records, (2) absence of either preoperative or postoperative computed tomography (CT) imaging, (3) follow-up shorter than 12 months, (4) incomplete/insufficient documentation of postoperative follow-up visits, and (5) absence of standardized pre- and postoperative photographic documentation.

For each patient meeting all eligibility requirements, the following materials and variables were retrieved and compiled into a structured dataset:

- Demographic data: sex and age
- Preoperative documents: surgical planning sheet, preoperative CT scan, and standardized preoperative photographs
- Intraoperative materials: operative report and intraoperative video recordings
- Postoperative documentation: operative clinical record, complete follow-up reports, postoperative CT scan, and standardized postoperative photographs

All extracted data were tabulated using Microsoft Excel for Mac, version 16.83 (Microsoft Corporation, Redmond, WA) to create a dedicated study dataset. Each patient was assigned a unique anonymous identification code, which was cross-referenced with the original records for data traceability while ensuring confidentiality. The final dataset used for analysis contained no personal identifiers. Deformity types were determined by reviewing preoperative CT scans and clinical photographs. I-shaped deviations were defined as linear lateral shifts of the nasal axis, C-shaped as uniplanar concave–convex curves, and S-shaped as complex, bidirectional curvatures involving both bony and cartilaginous components.^6^ These categories were used to perform subgroup analysis of outcomes, including residual deviation and satisfaction scores.

### Surgical Technique

All procedures were performed in an accredited operating theater under monitored intravenous sedation (maintaining spontaneous breathing) combined with local infiltration anesthesia. Local anesthesia consisted of 2% mepivacaine with epinephrine 1:100,000.

### Inferior Turbinate Management

In all patients, bipolar electrocauterization of the inferior turbinates was performed at the beginning of the procedure to improve nasal airflow and minimize postoperative edema.^7^

The procedure followed a standardized sequence: septoplasty, dorsal bony correction, osteotomies, and finally tip refinement.

**Figure 1.**
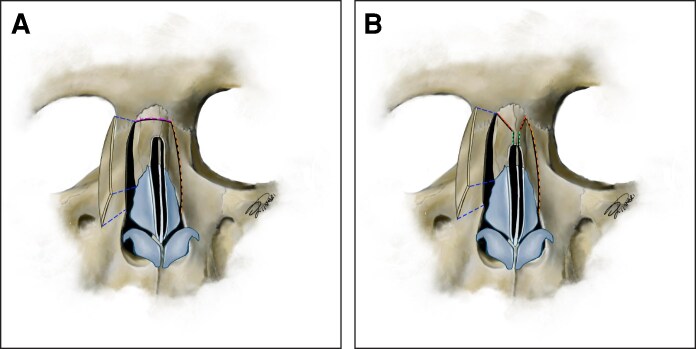
Pisa Tower–style asymmetric osteotomy configurations. (A) Schematic representation of the osteotomy configuration used when deviation originates from the radix. A high transverse osteotomy at the radix is performed (purple line) to allow en bloc mobilization and realignment of the nasal bones. This is combined with wedge resection on the convex (long) side (blue dashed lines) and a lateral osteotomy on the concave (short) side (orange dashed line). (B) Schematic representation of the osteotomy configuration used when deviation originates below the radix or involves the midvault. A paramedian osteotomy (green line) followed by an oblique osteotomy (red line) is performed to allow segmental mobilization and narrowing of the bony pyramid. This is combined with wedge resection on the convex (long) side (blue dashed lines) and a basal lateral osteotomy on the concave (short) side (orange dashed line).

1. Septoplasty: A structured open rhinoplasty was performed using a transcolumellar incision combined with bilateral marginal incisions. Elevation of the soft tissue envelope was conducted in a sub-SMAS (The Superficial Musculoaponeurotic System) plane to ensure preservation of vascular supply and minimize trauma (Video 1, available online at https://doi.org/10.1093/asjof/ojag064). An endoscope-assisted septoplasty followed, with meticulous subperichondrial and subperiosteal dissection to expose the cartilaginous and bony septum (Video 2, available online at https://doi.org/10.1093/asjof/ojag064). Deviated segments of the septal cartilage and vomer were resected while preserving a stable L-strut with a minimum width of 1.5 cm in both the dorsal and caudal limbs (Video 2).^8^ Resection of the dorsal septal cartilage was performed when necessary to improve dorsal alignment and avoid contour irregularities. When asymmetry or instability of the upper lateral cartilages (triangular cartilages) was identified, they were either converted into spreader flaps or resected and reconstructed with spreader grafts harvested from autologous septal cartilage.^9^ These steps ensured restoration of the internal nasal valve angle and preservation of harmonious dorsal aesthetic lines.
2. Dorsal Reduction: Reduction of the osseocartilaginous hump was performed in a stepwise manner. First, a 4 mm straight osteotome was used for controlled resection of the bony dorsum, followed by fine rasping to eliminate sharp edges and achieve symmetry. The cartilaginous hump was then resected under direct vision, with care taken to preserve the scroll region and attachments of the upper lateral cartilages.^8^ This technique prevented disruption of the internal nasal valve and contributed to balanced dorsal aesthetic lines. When required, spreader grafts (Video 3, available online at https://doi.org/10.1093/asjof/ojag064) were positioned asymmetrically, preferentially on the concave side of the deviation, to stabilize the dorsal septum and restore midvault balance (Videos 4 and 5, available online at https://doi.org/10.1093/asjof/ojag064)
3. Osteotomies (Pisa Tower Concept): The osteotomy approach was selected based on the anatomical origin of the nasal deviation. In cases where the deviation originated from the radix, only a transverse osteotomy was performed high across the nasal root using a Cakir-style osteotome (Figure 1A). This instrument allows for precise, medial-oblique cuts that facilitate controlled separation of the nasal bones at the keystone area, enabling en bloc mobilization and symmetric realignment of the bony vault. Conversely, when the deviation originated below the radix or involved the midvault, an alternative configuration was adopted: The transverse osteotomy was omitted in favor of paramedian oblique or median oblique osteotomies, performed with a guarded osteotome to allow controlled inward movement and symmetric narrowing of the bony pyramid^10^ (Figure 1B). On the convex (long) side, a wedge resection of the ascending maxillary process was performed using a rongeur, with the bone thickness removed (typically 2-4 mm) tailored to the degree of deviation. Prior to resection, medial and lateral subperiosteal dissection of the bony sidewall was performed using a Freer elevator to facilitate mobilization. On the concave (short) side, a low-to-low or high-to-low to high lateral osteotomy was performed using a curved 2 mm guarded osteotome. This tailored approach ensured anatomical correction while minimizing the risk of over-narrowing or dorsal step-off (Videos 4-6, available online at https://doi.org/10.1093/asjof/ojag064).
4. Tip Reconstruction: A septal extension graft (SEG), harvested from native septal cartilage, was placed to provide structural support and long-term projection of the nasal tip. The lower lateral cartilages were reshaped and stabilized using interdomal sutures and, when needed, reinforced with shield grafts. Particular attention was paid to preserving and reattaching the Pitanguy ligament to the dermocartilaginous junction.^11^ This maneuver contributed to natural tip dynamics, optimized soft tissue redraping, and helped prevent postoperative supratip fullness (Video 7, available online at https://doi.org/10.1093/asjof/ojag064)

This study was conducted in accordance with the principles of the Declaration of Helsinki. IRB approval was obtained from the Ethics Committee of the Santa Rita Clinic, Avellino, Italy. All patients provided informed written consent for surgical procedures, data analysis, and publication of clinical data and images. This retrospective study follows the Strengthening the Reporting of Observational Studies in Epidemiology (STROBE) guidelines.

### Representative Case Selection

As part of the photographic documentation included in the study dataset, 3 clinical cases were selected for illustrative purposes. These cases reflect the spectrum of crooked nose deformities encountered—namely I-shaped, C-shaped, and S-shaped—and were chosen to demonstrate the applicability and outcome consistency of the Structural Pisa Tower technique. All selected cases had complete pre- and postoperative standardized photographic documentation and follow-up exceeding 12 months.

### Outcome Measures

Surgical outcomes were assessed using clinical, radiological, and patient-reported measures. Photographic analysis was used to quantify external alignment by measuring the nasal axis deviation angle on standardized frontal photographs obtained with a fixed camera-to-subject distance, constant lighting, and the patient in natural head position.

Radiological assessment was performed on coronal CT images by measuring the R–Webster distance, defined as the horizontal deviation of the rhinion from the facial midline. Prior to measurement, each CT volume was re-oriented on the frontal plane: The horizontal distance from the lateral orbital rim to a vertical line passing through the most lateral point of the zygomatic arch was measured bilaterally, and the dataset was rotated until these 2 distances were equal to ensure symmetrical craniofacial alignment. Measurements were performed using RadiAnt DICOM Viewer (Medixant, Poznań, Poland; version 2025.2) and reported in millimeters. Postoperative CT imaging was not routinely performed for aesthetic rhinoplasty; in this series, it was selectively obtained in patients with preoperative skeletal deviation > 5° or when radiologic confirmation of bony vault realignment was required for study purposes, in order to limit unnecessary radiation exposure.

Patient satisfaction was assessed at final follow-up using a 5-point Likert scale (1 = very dissatisfied; 5 = very satisfied). Functional outcomes were evaluated using the validated Italian version of the Nasal Obstruction Symptom Evaluation (I-NOSE) questionnaire (range 0-100; higher scores indicate worse obstruction), administered preoperatively and at the final postoperative follow-up. When baseline data were unavailable, a retrospective pretest I-NOSE score was collected at follow-up, based on the patient's recalled preoperative breathing status. In addition, patients rated perceived functional change using a 5-point Likert anchor (1 = much worse; 5 = much better vs preoperative status).

All photographic and CT-based measurements were independently performed by 2 authors (S.D.A. and D.D.C.), blinded to each other's results. CT evaluations were double-checked by an experienced radiologist to ensure consistency. Any discrepancies exceeding 0.5 mm or 0.5° were jointly reviewed, and the mean value was recorded for analysis.

### Complication Assessment and Follow-Up

All intraoperative and postoperative records were systematically reviewed to identify adverse events. Two authors (S.D.A. and D.D.C.) screened the operative report, anesthesia record, immediate recovery notes, and every outpatient follow-up entry available up to the last documented visit (minimum 12 months, maximum 28 months). Discrepancies were resolved by discussion, and final validation was given by the senior author (N.B.)

Complications were categorized as follows:

- Minor—events not requiring additional surgery (eg, persistent edema > 3 months, rocker deformity without functional impact, and mild residual deviation).
- Major—events necessitating unplanned surgical revision due to significant morbidity (eg, infection, skin necrosis, and nasal valve collapse).

Relevant complication data for each patient were extracted and incorporated into the final dataset.

### Statistical Analysis

Data were tabulated in Microsoft Excel (Microsoft Corporation; version 16.0) and analyzed in R, version 4.4.0 (R Foundation for Statistical Computing, Vienna, Austria). Continuous variables are presented as mean ± SD and range and categorical variables as absolute counts and percentages. The normality of continuous distributions (nasal axis angle and R–Webster distance) was assessed with the Shapiro–Wilk test. Preoperative vs postoperative values were compared using a paired Student's *t* test. Patient satisfaction scores and postoperative complications were summarized descriptively. A *P*-value of <.05 was considered statistically significant.

Although patients were categorized into 3 morphological subtypes to reflect increasing anatomical complexity, inferential comparisons between groups were not performed due to limited sample size. Descriptive stratification was instead adopted to contextualize outcomes by deformity pattern. Pre- and postoperative I-NOSE scores were compared using a paired *t* test. Postoperative I-NOSE and Likert scores were summarized as means and proportions. Statistical significance was set at *P* < .05.

## RESULTS

A total of 43 cases were initially screened; 6 were excluded due to incomplete documentation or insufficient follow-up, leaving 37 patients for final analysis.

### Patient Demographics

The study included 37 patients (29 females, 8 males) with a mean age of 31.7 ± 8.9 years (range 18-52). All underwent structural rhinoplasty with asymmetric Pisa Tower–style osteotomies performed by a single surgeon. Deviation patterns were classified as I-shaped in 10 patients (27%), C-shaped in 15 (41%), and S-shaped in 12 (32%). Preoperative CT was available for all patients and postoperative CT for 35 (94.6%). No patient was lost to follow-up (Table 1). The mean follow-up was 18 months (range 12-28 months).

**Table 1.** Demographic and Clinical Characteristics of the Study Population

Variable	*n* (%)/Mean ± SD (range)
Total patients	37
Sex	29 female (78.4%), 8 male (21.6%)
Age (years)	31.7 ± 8.9 (18-52)
Type of deviation	I-shaped = 10 (27%) C-shaped = 15 (41%) S-shaped = 12 (32%)
Follow-up (months)	18 ± 5 (12-28)
Preoperative CT available	37 (100%)
Postoperative CT available	35 (94.6%)
Lost to follow-up	0 (0%)

Summary of baseline demographic and clinical characteristics for the 37 patients included in the study.

CT, computed tomography; SD, standard deviation.

Photographic assessment revealed a significant improvement in nasal axis deviation, from 7.9 ± 1.9° (range 5.2-11.3°) preoperatively to 0.9 ± 0.8° (range 0.3-2.6°) postoperatively (*P* = .0001).

In contrast, radiological evaluation based on coronal CT images showed that the R–Webster distance on the convex side decreased from 30.4 ± 4.8 mm (range 24-42 mm) to 23.1 ± 3.9 mm (range 18-31 mm) (*P* = .0003). On the concave side, the distance decreased from 23.7 ± 3.5 to 22.9 ± 3.3 mm (*P* = .18) (Table 2). When stratified by deformity pattern, the mean postoperative deviation was 0.7° for I-shaped, 1.0° for C-shaped, and 1.9° for S-shaped noses.

**Table 2.** Radiologic Outcomes of Nasal Realignment: Comparison of Preoperative and Postoperative Values of Nasal Axis Deviation and R–Webster Angle on Both the Convex and Concave Sides, Demonstrating the Effectiveness of Asymmetric Osteotomies Based on the Pisa Tower Concept

Measurement	Preoperative (mean ± SD, range)	Postoperative (mean ± SD, range)	*P*-value
Nasal axis deviation (°)	7.9 ± 1.9 (5.2-11.3)	0.9 ± 0.8 (0.3-2.6)	.0001
R–Webster distance (convex side, mm)	30.4 ± 4.8 (24-42)	23.1 ± 3.9 (18-31)	.0003
R–Webster distance (concave side, mm)	23.7 ± 3.5 (18-30)	22.9 ± 3.3 (17-29)	.18

SD, standard deviation.

The greatest correction was achieved in I-shaped deviations, while S-shaped noses demonstrated higher variability, with 2 of 12 patients showing residual asymmetry > 2.0° despite technically adequate correction. Patient satisfaction mirrored these findings, with “very satisfied” responses reported by 72% of I-shaped, 66% of C-shaped, and 43% of S-shaped patients (Table 3).

**Table 3.** Outcomes by Deformity Type

Deviation type	*N*	Mean postoperative deviation (°)	Residual asymmetry > 2°	Very satisfied (%)
I-shaped	18	0.7 ± 0.3	0/18	∼72%
C-shaped	12	1.0 ± 0.5	1/12	∼66%
S-shaped	7	1.9 ± 0.8	2/7	∼43%

Postoperative nasal deviation and satisfaction rates stratified by I-, C-, and S-shaped deformities.

### Comparison by Deviation Type

Subgroup analysis based on deviation pattern (I-, C-, or S-shaped) revealed no statistically significant difference in postoperative R–Webster improvement (*P* = .29, 1-way analysis of variance [ANOVA]). However, the mean residual deviation was slightly higher in C- and S-shaped cases, consistent with their greater preoperative asymmetry.

The choice of osteotomy configuration was determined by the anatomical level of deviation rather than its morphologic type. Transverse osteotomies were predominantly used for I-shaped and upper-third deviations (8 of 10 cases), whereas paramedian or oblique osteotomies were more frequent in C- and S-shaped noses (20 of 27 cases). Statistical analysis showed no significant correlation between deviation shape and osteotomy type (*P* = .33, chi-square test), confirming that the surgical plan was individualized according to structural anatomy rather than deviation morphology.

**Figure 2.**
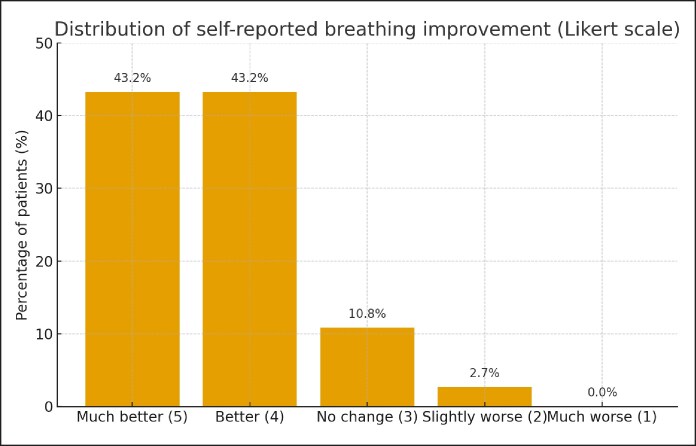
Distribution of patient-reported satisfaction scores assessed at final follow-up using a 5-point Likert scale (1 = very dissatisfied; 5 = very satisfied). Overall, 89.2% of patients reported high satisfaction (scores 4-5) at a mean follow-up of 18 months (range 12-28 months).

At final follow-up (mean 18 months), 24 patients (64.9%) rated their result as 5 on the 5-point Likert scale, 9 (24.3%) as 4, 3 (8.1%) as 3, and 1 (2.7%) as 2. No patients reported complete dissatisfaction (score 1), resulting in an overall satisfaction rate of 89.2% (Table 4; Figure 2).

**Table 4.** Patient Satisfaction (Likert Scale)

Score	No. of patients	Percentage
5—Very satisfied	24	64.9%
4—Satisfied	9	24.3%
3—Neutral	3	8.1%
2—Dissatisfied	1	2.7%
1—Very dissatisfied	0	0%

Distribution of patient satisfaction scores 12 months postoperatively using a validated 5-point Likert scale assessing aesthetic outcomes.

Minor postoperative complications occurred in 5 patients (13.5%), including 2 cases of persistent edema beyond 3 months, 1 rocker deformity not requiring correction, 1 case of residual deviation, and 1 elective minor revision for a localized dorsal irregularity, performed under local anesthesia and not associated with functional impairment. No major complications—such as infection, cutaneous necrosis, or nasal valve collapse—were observed (Table 5).

**Table 5.** Postoperative Complications

Complication	No. of patients	
Persistent edema > 3 months	2	5.4%
Rocker deformity (nonvisible)	1	2.7%
Residual deviation	1	2.7%
Revision for dorsal irregularity	1	2.7%
Major complications (infection, necrosis, valve collapse)	0	0%

Overview of minor and major postoperative complications observed during the follow-up period, including persistent edema, deformities, and surgical revisions.

### Functional Outcomes

Pre- and postoperative I-NOSE data were available for 30 of the 37 patients (81%). The mean preoperative I-NOSE score was 56.4 ± 14.8, decreasing significantly to 18.9 ± 11.3 at final follow-up. In the 7 patients without baseline data, the retrospective pretest I-NOSE averaged 59.7 ± 13.2 compared with a postoperative mean of 20.1 ± 10.5 (*P* = .0001). On the 5-point Likert anchor, 32 of 37 patients (86.5%) reported improved nasal breathing (“better” = 16; “much better” = 16), 4 (10.8%) reported “no change,” and 1 (2.7%) reported “slightly worse.” Overall, the mean relative reduction in I-NOSE score was 66.5%, consistent with clinically meaningful improvement in perceived nasal airflow (Table 6).

**Table 6.** Functional Outcomes (I-NOSE)

Parameter	No. of patients (*n* = 37)	Preoperative (mean ± SD)	Postoperative (mean ± SD)	Mean change	*P*-value
I-NOSE score (0-100)^a^	30 (81%)	56.4 ± 14.8	18.9 ± 11.3	−37.5 ± 13.6 (−66.5%)	.00002
Retrospective pretest I-NOSE^b^	7 (19%)	59.7 ± 13.2	20.1 ± 10.5	−39.6 ± 12.9 (−66.3%)	.0001

^a^I-NOSE, Italian Nasal Obstruction Symptom Evaluation; higher scores indicate greater obstruction. bRetrospective pretest administered at follow-up (“as recalled preoperative condition”).

### Representative Clinical Cases

To illustrate the application and outcomes of the Structural Pisa Tower technique, 2 representative cases of patients with crooked nose deformities are presented. These examples were selected to reflect the anatomical variability encountered in clinical practice and the adaptability of the proposed surgical strategy.

#### Case 1

**Figure 3.**
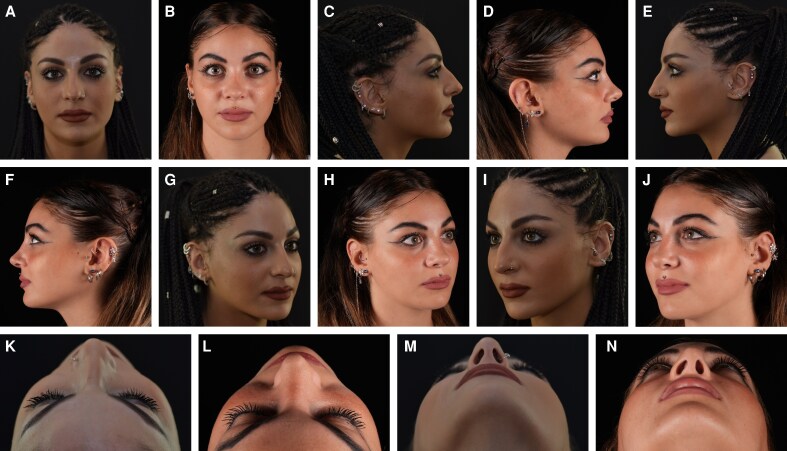
Standardized clinical photographs of a 30-year-old female patient with a C-shaped nasal deviation (Case 1) with (A, C, E, G, I, K, L, and M) preoperative and (B, D, F, H, J, L, and N) and 24-month postoperative views.

A 30-year-old female patient presented with a C-shaped nasal deviation involving the bony nasal vault. Preoperative standardized photographs (Figure 3A-G) demonstrated axial deviation with asymmetry of the dorsal aesthetic lines on frontal, oblique, and basal views. Correction was achieved through asymmetric lateral osteotomies, consisting of medialization on the concave side and controlled shortening of the convex side. A transverse osteotomy at the radix was performed to allow mobilization and realignment of the nasal bones. Septal correction and dorsal refinement were completed according to the operative protocol.

**Figure 4.**
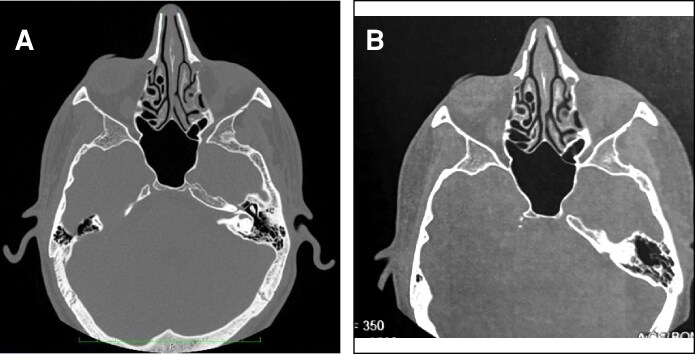
(A) Representative preoperative axial computed tomography image of Case 1, obtained during preoperative planning. (B) Representative postoperative axial computed tomography image of Case 1, obtained at final follow-up (24 months). Images are provided to qualitatively illustrate bony vault realignment following Pisa Tower–style asymmetric osteotomies.

At 24-month follow-up, postoperative photographs (Figure 3H-N) showed improved nasal axis alignment, symmetric dorsal aesthetic lines, and balanced nasal base appearance. Representative axial CT images (Figure 4A, B) qualitatively confirmed bony vault realignment at final follow-up.

#### Case 2

**Figure 5.**
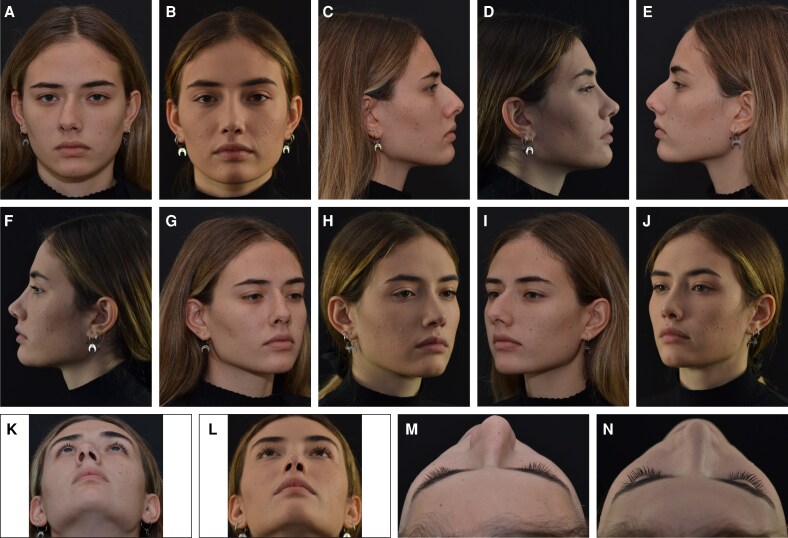
Standardized clinical photographs of a 27-year-old female patient with an I-shaped nasal deviation (Case 2) with (A, C, E, G, I, K, L, and M) preoperative and (B, D, F, H, J, L, and N) and 18-month postoperative views.

A 27-year-old female patient presented with an I-shaped deviation and minimal cartilaginous irregularity. Preoperative photographs (Figure 5A-G) show linear axis deviation without dorsal hump. At 18-month follow-up (Figure 5H-N), frontal and basal views reveal symmetric correction and balanced tip projection. In this patient, correction was achieved through a low-to-low lateral osteotomy on the concave side and a limited paramedian oblique osteotomy for segmental midvault adjustment, without the need for a transverse cut at the radix. Photographic measurement showed a deviation of 6.8° preoperatively and 0.6° postoperatively. CT evaluation revealed a reduction in R–Webster distance from 29.0 to 22.9 mm on the convex side and from 23.5 to 22.7 mm on the concave side. These representative cases illustrate the practical application of the Pisa Tower–style structural technique in different types of nasal deviation, highlighting the consistency of postoperative correction and aesthetic symmetry observed across the cohort.

## DISCUSSION

This study demonstrates that asymmetric osteotomies inspired by Finocchi's Pisa Tower Concept can be successfully adapted to a structural rhinoplasty framework. While the original concept was developed within preservation rhinoplasty, aiming to maintain the dorsal roof intact, our adaptation integrates its geometric principles into a structural workflow that includes dorsal hump reduction, endoscopic septoplasty, and tip reconstruction. This structural reinterpretation allows the technique to be applied to more complex crooked noses requiring extensive skeletal realignment and septal correction.

To our knowledge, this is the first study to apply Pisa Tower–style osteotomies within a structural rhinoplasty workflow, combining dorsal hump management, endoscopic L-strut septoplasty, and tip reconstruction with septal extension grafts. This adaptation expands the technique's applicability to more complex deviations involving both bony and cartilaginous components.

The significant reduction in nasal axis deviation and R–Webster angles validates the geometric rationale of asymmetric osteotomy planning. By differentially mobilizing the convex and concave nasal walls, this method enables precise 3D correction and realignment of the bony vault. Although these measurements are primarily radiological, they correlate well with visual symmetry and dorsal contour alignment, supporting their clinical relevance in postoperative evaluation.

When outcomes were stratified by deformity pattern, the technique appeared most effective in I-shaped deviations, which showed the most consistent radiological realignment (mean postoperative deviation 0.7°, 0% residual asymmetry). C-shaped deformities also responded well, although mild asymmetry persisted in 1 of 12 patients. S-shaped deviations exhibited the greatest variability, with 2 of 12 patients showing residual asymmetry > 2.0°. This reflects anatomical limitations commonly associated with multilevel curvature, which may require more aggressive remodeling, such as extracorporeal septoplasty or the use of multiple grafts for long-term midvault stability. Consistent with previous literature, C- and S-shaped deviations showed slightly higher residual asymmetry compared with I-shaped noses.^12^ This may reflect the intrinsic difficulty of fully correcting multilevel deformities and the limited modulation of the keystone area achievable with structural osteotomies alone.

This limitation becomes particularly relevant when dorsal deviation extends beyond the nasal bones into the cartilaginous keystone area. Unlike preservation approaches that maintain the osseocartilaginous vault as a single unit—allowing controlled repositioning of the keystone complex—the structural Pisa Tower technique relies on discrete osteotomies and septal reconstruction, which offer less flexibility for en bloc dorsal realignment. Consequently, minor residual asymmetries in C- and S-shaped noses may arise from this inherent geometric constraint rather than from technical inaccuracy.

Statistical subgroup analysis confirmed that postoperative R–Webster improvement did not significantly differ among deviation types (*P* = .29, 1-way ANOVA). However, residual deviation tended to be higher in C- and S-shaped noses (mean 1.8 ± 0.6 mm and 2.1 ± 0.7 mm, respectively) compared with I-shaped cases (1.4 ± 0.5 mm), consistent with their greater preoperative asymmetry.

These findings suggest that although asymmetric Pisa Tower–style osteotomies achieve substantial correction across all deformity patterns, complex multilevel deviations remain more prone to residual asymmetry, largely due to limited modulation of the keystone area achievable through bony osteotomies alone.

Several structural rhinoplasty strategies have been proposed to correct crooked noses, typically using symmetric low-to-low or low-to-high osteotomies combined with septal realignment and spreader graft stabilization. Toriumi and Most emphasized balanced osteotomies with rigid midvault reconstruction to restore both aesthetics and function,^13,14^ whereas Pearlman and Haack recently described hybrid preservation-structural approaches to minimize dorsal irregularities and maintain keystone stability.^10^

In contrast, the Pisa Tower–style technique introduces a deliberate geometric asymmetry—combining wedge resection on the convex side with controlled push-down on the concave side—to achieve targeted correction while avoiding excessive dorsal widening. This asymmetric planning provides greater adaptability in cases of unilateral vault deviation and complements existing structural techniques rather than replacing them. Compared to classic bilateral osteotomies and structural frameworks relying on symmetric mobilization, our approach showed greater adaptability in addressing unilateral or asymmetric vault deviations.

The selection of transverse vs paramedian or oblique osteotomies was anatomy driven, depending on the level of deviation origin rather than the external deviation pattern. I-shaped deformities benefited most from transverse osteotomies at the radix, whereas C- and S-shaped cases typically required oblique or paramedian cuts for accurate segmental realignment. Similar to hybrid strategies described by Toriumi and Most, the use of tailored osteotomies and segmental septal correction enabled a more anatomy-specific correction of the nasal framework.^5^

Traditional bilateral low-to-low osteotomies often inadequately address asymmetrical deformities, particularly when one side of the nasal vault is more pronounced or resistant.^15-17^ The asymmetric approach we employed—wedge resection on the convex side, paired with low-to-low or low-to-high or high-to-low-to-high osteotomies on the concave side—provides anatomically tailored mobilization while preserving dorsal width. These principles mirror those of asymmetric dorsal preservation techniques, which have shown success in I-shaped deformities.^18^ The option to add either a transverse osteotomy at the radix or paramedian/median oblique osteotomies at the midvault—selected based on deformity origin—adds flexibility. Transverse osteotomy allows en bloc mobilization of the nasal bones when deviation originates from the root, while oblique cuts enable segmental realignment for midvault deformities, optimizing anatomical correction and nasal stability. The consistent use of endoscopic-assisted L-strut septoplasty enhanced surgical precision and septal support. This method preserves mucoperichondrial flaps and reduces postoperative edema, paralleling best practices described in structural rhinoplasty literature.^19^ Tip stability was reinforced with septal extension grafts, reshaped lower lateral cartilages, interdomal sutures, and preservation of the Pitanguy ligament. Anatomic studies confirm the ligament's role in tip support, rotation, and prevention of supratip deformity.^20,21^ Use of CT imaging to objectively measure bone realignment lends scientific rigor, though it contrasts with the increasing preference for reduced-radiation methods. Nevertheless, in complex deformities, CT remains the gold standard for bony assessment.^19^

Although CT imaging enabled objective measurement of bony deviation and surgical correction, its routine use in aesthetic follow-up may raise concerns. While justified here for complex anatomical analysis, CT may not be cost-effective or ethically sustainable for all patients, particularly given cumulative radiation exposure. Our 13.5% minor complication rate aligns with outcomes from experienced centers performing structural rhinoplasty,^22^ and the absence of major adverse events reaffirms procedural safety. Patient satisfaction, with nearly 90% rating 4 to 5 on the Likert scale, compares favorably with outcomes reported in the structural and hybrid rhinoplasty literature.^18,22^ In contrast to Finocchi's preservation-only series, our work applies Pisa Tower geometry within a structural paradigm, combining dorsal reduction, septal stabilization, and tip refinement. This integration broadens the technique's clinical relevance, especially in cases requiring greater control over internal architecture.^3^

Functional analysis using the validated I-NOSE questionnaire demonstrated a significant postoperative improvement, with mean scores decreasing from 56 to 19, confirming that asymmetric correction of the bony vault also translated into subjective respiratory benefit. Although the evaluation relied on patient-reported outcomes without objective rhinomanometry, the use of a validated patient reported outcome strengthens the clinical relevance of the findings.

This study is limited by its retrospective design, single-surgeon experience, and relatively small cohort size, which restricts the statistical power and generalizability of the findings. Although all patients underwent standardized photographic documentation, subtle measurement variability may have occurred due to minor differences in head positioning or lighting conditions. Similarly, despite careful CT reorientation before analysis, minimal discrepancies in plane alignment could have influenced millimetric accuracy. Functional assessment also presents limitations: While the I-NOSE questionnaire provided validated subjective data, not all patients completed it prospectively, and no objective rhinomanometric measurements were performed.

Finally, the absence of a control group prevents direct comparison with traditional symmetric osteotomy techniques. Future prospective studies with larger patient populations, standardized photographic and radiologic protocols, and objective airflow testing are warranted to confirm the reproducibility and long-term stability of the Pisa Tower–style structural approach.

## CONCLUSIONS

This preliminary experience suggests that Pisa Tower–style asymmetric osteotomies *may provide* predictable and stable outcomes across different nasal deviation patterns, though further validation in larger cohorts is needed. The technique combines Finocchi's asymmetric geometric rationale with structural dorsal and septal reconstruction, demonstrating reproducible aesthetic and functional improvement in crooked nose deformities.
